# Mechanistic understanding of asphaltene precipitation and oil recovery enhancement using SiO_2_ and CaCO_3_ nano-inhibitors

**DOI:** 10.1038/s41598-024-65995-1

**Published:** 2024-07-02

**Authors:** Ali Shadervan, Arezou Jafari, Alireza Teimouri, Reza Gharibshahi, Amir Hossein Saeedi Dehaghani

**Affiliations:** Faculty of Chemical Engineering, Tarbiat Modares University, Tehran, Iran

**Keywords:** Nano-inhibitor, Asphaltene, Onset, Oil recovery, Micromodel, Chemical engineering, Nanoparticles

## Abstract

Asphaltene precipitation in oil reservoirs, well equipment, and pipelines reduces production, causing pore blockage, wettability changes, and decreased efficiency. Asphaltenes, with their unique chemical structure, self-assemble via acid–base interactions and hydrogen bonding. Nano-inhibitors prevent asphaltene aggregation at the nanoscale under reservoir conditions. This study investigates the effect of two surface-modified nanoparticles, silica, and calcium carbonate, as asphaltene inhibitors and oil production agents. The impacts of these nano-inhibitors on asphaltene content, onset point, wettability, surface tension, and oil recovery factor were determined to understand their mechanism on asphaltene precipitation and oil production. Results demonstrate that these nano-inhibitors can significantly postpone the onset point of asphaltene precipitation, with varying performance. Calcium carbonate nano-inhibitor exhibits better efficiency at low concentrations, suspending asphaltene molecules in crude oil. In contrast, silica nano-inhibitor performs better at high concentrations. Wettability alteration and IFT reduction tests reveal that each nano-inhibitor performs optimally at specific concentrations. Silica nano-inhibitors exhibit better colloidal stability and improve oil recovery more than calcium carbonate nano-inhibitors, with maximum oil recovery factors of 33% at 0.1 wt.% for silica and 25% at 0.01 wt.% for calcium carbonate nano-inhibitors.

## Introduction

Asphaltenes are one of the complicated components of reservoir oil that are soluble in benzene or toluene and insoluble in light paraffin, such as n-pentane or n-heptane^1^. Asphaltene precipitation and deposition are significant reservoir problems, reducing productivity and oil recovery^2,3^. This issue is caused by changes in pressure, temperature, and oil compositions^4,5^. Pore plugging, permeability reduction, decreasing efficiency of the facilities, and wettability alteration are drawbacks of asphaltene precipitation and deposition^6–8^. Two leading solutions for addressing this problem are removing precipitated asphaltene or utilizing inhibitors to delay this problem. However, the first method is too expensive and time-consuming. Thus, stabilizing asphaltene in oil reservoirs has been considered by many scientists^9,10^.

Recently, nanoparticles have been introduced as novel materials for solving asphaltene precipitation and also improving oil recovery because of their surface properties and charges^11,12^. Mehrooz et al. introduced a novel method for synthesizing cerium oxide nanoparticles (in-situ synthesis) and demonstrated its enhanced oil recovery potential. However, its impact on asphaltene precipitation inhibition remains unexplored^13^. Hashemi et al. examined the influence of nickel oxide nanoparticles on asphaltene precipitation destabilization in the presence of carbon dioxide^14^. Ahmadi et al. focused on the use of nickel–zeolite oxide to explore the relationship between asphaltene adsorption on the surface of nickel–zeolite oxide nanoparticles and asphaltene precipitation in the presence of nanoparticles during the actual process. They demonstrated that monolayer adsorption of asphaltene occurred on the surface of nickel–zeolite oxide nanoparticles using the Langmuir model isotherm, resulting in a reduction of asphaltene precipitation. Additionally, they found that decreasing the pressure from 1650 to 1350 psi increased asphaltene precipitation from 8.25 to 10.52 wt.%^15^.

Mansouri et al. conducted asphaltene precipitation tests in a static phase and dynamic CO_2_ flooding tests in the presence of zeolite‑zirconia‑copper nanocomposites, comparing the results with those for zeolite nanoparticles. The results showed that zeolite‑zirconia‑copper nanocomposites had a lower surface area, higher pore volume, and larger diameter compared to zeolite nanoparticles. The efficiency of zeolite‑zirconia‑copper nanocomposites in adsorbing asphaltene on their surface and decreasing asphaltene precipitation was higher than that of zeolite nanoparticles. Reducing the pressure from 2600 to 1700 psi caused asphaltene precipitation in the presence of zeolite‑zirconia‑copper nanocomposites to decrease from 5.10 to 2.80 wt.% and 3.78 wt.%, respectively. In the presence of zeolite, asphaltene precipitation decreased from 14.24 to 6.25 wt.% and 8.40 wt.%, respectively. Furthermore, in CO_2_ dynamic tests, it was observed that zeolite‑zirconia‑copper nanocomposites had high potential for improving permeability impairment, reducing porosity, and decreasing the asphaltene deposition rate^16^. Li et al. investigated the effectiveness of three nanoparticle types—NiO, SiO_2_, and Fe_3_O_4_—in inhibiting asphaltene precipitation in water-wet porous media, attributing their efficacy to their high surface area-to-volume ratio, strong adsorption capacity, and excellent suspension characteristics^17^. Based on DLVO theory, surface charges of nanoparticles and asphaltenes polarity can have some interactions, leading to the stability of asphaltene^18^. The effect of various nanoparticles, including NiO, Fe_3_O_4,_ and γ-Al_2_O_3_ nanoparticles, on suppressing asphaltene precipitation was evaluated by Shojaat et al.^19^. They concluded that γ-Al_2_O_3_ nanoparticles at 0.1 wt.% exhibited the best efficiency among these nanoparticles. Mohammadi et al. investigated the performance of the TiO_2_/SiO_2_ nanocomposites on the increment of the onset point of asphaltene^20^. They deduced that these nanocomposites’ high stability and surface area contribute to postponing asphaltene precipitation. Castillo et al. studied the adsorption of asphaltenes molecules on silica (SiO_2_) nanoparticles using GPC-ICP HR-MS^21^. They reported that Van der Waals forces are the primary interaction between these nanoparticles and asphaltene that adsorb asphaltene molecules and decline asphaltene aggregation.

The formation of asphaltene precipitation affects enhanced oil recovery (EOR) in two different ways. Firstly, it blocks available voids in porous media and limits flow paths for displacing oil. In other words, due to pore plugging by asphaltene precipitation, reservoir rock's effective permeability reduces significantly, negatively impacting oil recovery. Secondly, suppose the asphaltene content in crude oil exceeded and increased significantly. In that case, it tends to be adsorbed on the surface of a rock, and the wettability of reservoir rock shifts toward strongly oil-wet. Thus, there is an essential direct relation between asphaltene precipitation and EOR, which is necessary to pay attention to both of these fields of study simultaneously when a fluid is injected into the reservoir.

Many studies have been carried out to investigate the influence of nanoparticles on the wettability alteration of the reservoir rock^22–24^. Jang et al. investigated the impact of modified SiO_2_ nanoparticles with GPTMS (3-Glycidoxypropyl) trimethoxysilane, on the wettability alteration of dolomite and limestone rocks^25^. They claimed that strongly oil-wet rock surfaces could be altered to natural-wet and water-wet for dolomite and limestone rocks by utilizing the functionalized SiO_2_ nanoparticles. The influence of γ-Al_2_O_3,_ MgO, and TiO_2_ nanoparticles on reducing the contact angle between oil drop and carbonate rock surface in the presence of CO_2_ was examined by Nowrouzi et al.^26^. They revealed that TiO_2_ nanoparticles could reduce the contact angle between oil drops and carbonate rock's surface better than other nanoparticles. In the latest research, Keykhosravi et al. explored the impact of SiO_2_ nanofluid on the wettability alteration of carbonate rock from oil-wet to water-wet state^27^. They inferred that the wettability alteration of carbonate rock by SiO_2_ nanoparticles is time-dependent. After seven days, the lowest contact angle between the oil and carbonate thin sections was achieved at 0.3 wt.%.

Another crucial method to enhance oil recovery is declining interfacial tension (IFT) between water and oil. By altering interfacial properties, nanoparticles can effectively impact IFT reduction^28,29^. The effect of functionalized ZrO_2_ nanoparticles on IFT reduction between water–oil and water–air systems was studied by Esmaeilzadeh et al.^30^. They elucidated that utilizing ZrO_2_ nanoparticles for IFT reduction in the water–oil system is more effective than the water–air system. Zandahvifard et al. investigated the influence of modified and bare Fe_3_O_4_ nanoparticles on IFT reduction between oil and carbonated water^31^. They concluded that functionalized Fe_3_O_4_ nanoparticles showed a far better effect on reducing IFT than uncoated Fe_3_O_4_ nanoparticles.

For reservoir porous media imitation and observation, the various methods combination of using nanoparticles for oil recovery and micromodel experiments have been considered by many researchers^31–34^. For instance, Cheraghian evaluated the improvement of oil recovery in the presence of TiO_2_ nanoparticles in the five-spot glass micromodel test. He claimed that ultimate oil recovery with nano-titanium dioxide in surfactant flooding was 4.89% higher than the injection of surfactant solution^35^. The main problem with using nanostructures for oil recovery is the agglomeration of nanoparticles, reducing pore size and oil permeability. Using nanoparticles in high concentrations can accelerate this phenomenon and reverse the oil recovery process.

Despite the numerous studies conducted, Table 1, which summarizes relevant research utilizing silica and calcium carbonate nanoparticles to prevent asphaltene precipitation, reveals a limited focus on concurrently investigating the multifunctional role of nanoparticles in both asphaltene precipitation inhibition and oil production processes. Additionally, the precise impact of calcium carbonate (CaCO_3_) nanoparticles, a potentially suitable compound due to its high compatibility with most oil reservoir rocks (carbonate rocks), has not been thoroughly determined in these processes. In this regard, this research is divided into two sections. In the first section, the surface of SiO_2_ and CaCO_3_ nanoparticles were modified to make them oil-wet using oleic acid. Then, the influence of these nanoparticles as nano-inhibitors on the onset point and postponement of asphaltene deposition was determined. In the second part, various analyzes were performed to investigate the mechanism of these nanoparticles in improving crude oil recovery. Therefore, the colloidal stability of these nanoparticles in the injection fluid was determined by quantitative and qualitative methods. Then, the effect of these nanoparticles on the mechanisms of crude oil production, including reduction of capillary force, reduction of surface tension, and wettability alteration, were investigated. Finally, the movement of these particles in the porous medium and the ability of these nanoparticles to increase the oil recovery factor were studied using a micromodel setup.

**Table 1:** Summary of silica and calcium carbonate nano-inhibitors studies.

Reference	Nanoparticles	Remark on nanoparticles	Highlights
Mohammadi et al. 2011^20^	TiO_2_ ZrO_2_ SiO_2_	Investigation the effect of nanoparticles for stabilizing asphaltene particles in oil Use of Oil titration method	The performance of TiO_2_ nanoparticles in acidic conditions for improving asphaltene stability is positive but in basic conditions is negative The inhibition mechanism of TiO_2_ nanoparticles is hydrogen bonds in an acidic medium In alkaline conditions, none of the nanoparticles can form a hydrogen bond and prevent the precipitation of asphaltenes
Hosseinpour et al. 2013^36^	NiO CaCO_3_ Fe_2_O_3_ WO_3_ MgO ZrO_2_	Investigation the effect of nanoparticles to adsorb asphaltenes from asphaltene–toluene solutions Use of Centrifugation followed by UV–vis spectroscopy of the supernatant liquid	Because of acidity and surface charge, the performance of nanoparticles for adsorbing asphaltenes is in the order of NiO > Fe_2_O_3_ > WO_3_ > MgO > CaCO_3_ > ZrO_2_
Betancur et al. 2016^37^	SiO_2_	Investigation the effect of particle size and surface acidity of silica nanoparticles on their interaction and adsorption of asphaltenes Use of UV–visible spectrophotometer, nanosizer, and core for dynamic tests	The presence of active sites on the surface of nanoparticles plays an essential role in the adsorption of asphaltene molecules. The bigger nanoparticle size, the less adsorption asphaltene molecules There is a direct relation between acidity and asphaltene adsorption
Amin and Nazar 2016^38^	SiO_2_ γ-Al_2_O_3_ TiO_2_	Investigation the effect of nanoparticle types, asphaltene types, nanoparticle-to-solution ratio and temperature on the adsorption size of asphaltenes Use of UV–visible spectrophotometer	Alumina and silica nanoparticles have the highest and lowest adsorption, respectively The temperature has no statistical significance Asphaltenes with high aromaticity tend to adsorb more onto nanoparticles
Ahmadi and Aminshahidy 2018^39^	CaO and SiO_2_	Investigation the effect of nanoparticle concentration on asphaltene precipitation Use of PVT cell In the presence of CO2 at different temperatures	CaO nanoparticle decreased asphaltene precipitation from (0.32 wt.%, 0.62 wt.%) to (0.096 wt.%, 0.214 wt.%) SiO_2_ nanoparticles decreased asphaltene from (0.56 wt.%, 1.10 wt.%) to (0.27 wt.%, 0.52 wt.%)
Li et al. 2018^17^	NiO SiO_2_ Fe_3_O_4_	Investigated the effect of nanoparticles on the inhibition of asphaltene particle aggregation in a water-wet micro-sized pore	Important factors for preventing asphaltene precipitation including high surface area to volume ratio, good adsorption capacity, and high nanoparticle stability
López et al. 2020^40^	Cardanol/SiO_2_ cardanol	Investigation the effect of cardanol/SiO2 nanocomposites on preventing asphaltene damage Use of core flooding test under reservoir condition Use of FTIR and DLS	The nanocomposites showed good performance in order to inhibit asphaltene precipitation Using nanocomposites improves oil recovery by more than 50% when compared to the scenario with asphaltene damage
Hosseini Dastgerdi et al. 2022^41^	SiO_2_ Polyacrylamide (PAM)	Investigation the effect of silica–polyacrylamide nanocomposite as asphaltene precipitation inhibitor Use of FTIR polarizing microscope and FESEM technique	By increasing nanocomposite concentration, asphaltene precipitation decreases It seems that by increasing nanocomposite surface heterogeneities, the level of asphaltene molecules adsorption increased and the level of asphaltene self-association reduced

## Asphaltene aggregation mechanisms

In general, there are two mechanisms for asphaltene precipitation: solubility and colloidal approaches. In the solubility approach, precipitation occurs due to thermodynamic changes^42,43^:

- **Pressure/temperature reduction:** When the pressure or temperature in the reservoir decreases during production, asphaltene molecules may become insoluble and precipitate out of the oil.
- **Mixing with other fluids:** The mixing of crude oil with incompatible fluids such as gas, water, or additives can induce asphaltene precipitation due to changes in composition and conditions.
- **Reservoir fluid composition:** Changes in the composition of the reservoir fluid can affect the stability of asphaltenes and lead to precipitation.

Another mechanism, known as the colloidal approach, involves asphaltene molecules coming together to form larger particles, a process known as asphaltene aggregation. This phenomenon results from complex interactions between asphaltene molecules and their environment^3,42^:

- **Van der Waals forces:** Asphaltenes, being large and complex molecules with numerous aromatic rings and aliphatic chains, experience significant attraction through van der Waals forces, including dispersion forces and dipole–dipole interactions. These forces become particularly influential at close distances, leading to the aggregation of asphaltene molecules. Asphaltenes exhibit anisotropic polarizability due to the inherent anisotropy of C–H and aromatic C–C bonds. This anisotropy extends to C–X bonds, where X is a heteroatom (N, O, or S). This effect is particularly pronounced in asphaltene and resin molecules with extensive aromatic regions. Consequently, the van der Waals interaction manifests as an anisotropic attractive force, playing a crucial role in the stacking behavior of these molecules. Specifically, for asphaltene and resin molecules containing planar aromatic regions, the van der Waals interaction significantly contributes to the aggregate's energy near the equilibrium intermolecular distance.
- **π–π interactions:** Aromatic rings present in asphaltene molecules can engage in π–π stacking interactions. These interactions occur between the π–electron clouds of adjacent aromatic rings, generating attractive forces that facilitate the aggregation of asphaltene molecules. These interactions are strongest when the aromatic rings are aligned parallel to each other.
- **Hydrogen bonding:** Asphaltene molecules contain polar functional groups such as hydroxyl (–OH), carboxyl (–COOH), and amine (–NH_2_) groups. Hydrogen bonding is a crucial contributor to asphaltene/resin aggregate formation. This interaction occurs between a hydrogen atom (H) bonded to a highly electronegative atom (like O) and an electron-rich atom (acceptor) in a nearby molecule. Primarily electrostatic, this interaction is driven by the inherent charges of the participating atoms, making it stronger than π–π interactions. H-bonding helps asphaltene and resin molecules stick together (aggregate) by forming connections at various points on the molecules. For a strong H-bond, two factors are crucial: a clear path and the proper angle. There shouldn't be any bulky groups (steric hindrance) blocking the H atom (donor) and the electron-rich atom (acceptor) from getting close enough to form a strong bond. Moreover, the orientation of the molecules (orientation dependence) should be at the right angles for a strong H-bond. If the angles are off, the H-bond will be weak and won’t significantly contribute to the aggregation process. The number and strength of H-bonds in asphaltene/resin aggregates depend on the specific chemical composition and shapes of the molecules involved. The complex chemistry of these heavy fractions suggests a wide variety of H-bonds. However, it’s important to note that H-bonding isn’t the only player. Other attractive forces between different parts of the asphaltene/resin molecules can also be strong and compete with H-bonding for dominance. For instance, large aromatic regions in these molecules might have different interaction patterns.
- **Nucleation and growth:** Asphaltene aggregation typically begins with the nucleation of small clusters of molecules, followed by their growth through the addition of more molecules. This process may involve mechanisms such as molecular rearrangement, coalescence, and further interaction with solvent molecules or other components of the asphaltene. Additionally, the desorption of resins from the asphaltene surface can lead to its precipitation. When the ratio of resin to asphaltene decreases, the amount of asphaltene precipitation increases. Resins contain lower amounts of heteroatoms such as sulfur, oxygen, and nitrogen, resulting in smaller cores and lower polarity. As a result, resins exhibit greater solubility and can surround asphaltene aggregates, thereby keeping them in their colloidal form^3,42^.

## Materials and methods

### Crude oil

A crude oil sample was provided from one of the Iranian oil reservoirs. In Tables 2 and 3, the properties of used crude oil and its compositional analysis are presented, respectively.

**Table 2:** Crude oil properties.

Properties	Value
MW (g/mol)	161.042
SG	0.856
API	33.8
Density (g/cm^3^) @60°F	0.95
viscosity (cp)	180
C_30_^+^	
MW (g/mol)	956.25
SG	0.925

**Table 3:** The results of compositional analysis showing the percent of existing components in the crude oil.

Component	Mole percentage (%)
N_2_	0.557
CO_2_	0.816
C_1_	28.337
C_2_	7.681
C_3_	5.713
i-C_4_	1.524
n-C_4_	6.131
i-C_5_	1.919
n-C_5_	4.182
C_6_	3.516
C_7_	5.305
p-C_8_	4.964
p-C_9_	3.845
p-C_10_	3.078
p-C_11_	2.183
p-C_12_	1.571
p-C_13_	1.387
p-C_14_	1.244
p-C_15_	0.987
p-C_16_	0.647
p-C_17_	0.585
p-C_18_	0.479
p-C_19_	0.360
p-C_20_	0.340
p-C_21_	0.316
p-C_22_	0.264
p-C_23_	0.250
p-C_24_	0.238
p-C_25_	0.144
p-C_26_	0.128
p-C_27_	0.115
p-C_28_	0.09
p-C_29_	0.065
C_30_^+^	11.038
Total	100

### Nanoparticles

In the first series of conducted experiments which belonged to the determination of asphaltene onset, surface modification of SiO_2_ (purity of 98%, size of 10–15 nm, Merck Co.), and CaCO_3_ (purity of 98%, size of 15–40 nm, Merck Co.) nanoparticles were carried out by oleic acid. In the second series of experiments, unmodified SiO_2_ and CaCO_3_ nanoparticles (commercial types) were used to evaluate the EOR impacts of nanoparticles. The general properties of all chemicals used in this research are presented in Table 4.

**Table 4:** The properties of chemicals used in the experiments.

Chemicals*	MW (g/mol)	Density (g/cm^3^)	Formula	Application	Company/country
Sodium hydroxide	40.01	2.16	NaOH	To make the porous medium oil-wet	Tetra-Chem/Iran
Methanol	32.04	0.79	CH_3_OH		Merck/Germany
Toluene	92.14	0.87	C_7_H_8_		
n-heptane	100.20	0.68	C_7_H_16_		
Silane	32.12	1.31	H_4_Si		Tetra-Chem/Iran
Oleic Acid	282.47	0.895	C_18_H_34_O_2_	Used for Surface modification of nanoparticles	Merck/Germany

*The purity of all chemicals was 99%.

### Measurement of asphaltene content in the crude oil sample

Measurement of asphaltene content in crude oil samples was carried out based on the IP-143 standard^44^. At first, 5 g of crude oil was poured into the flask, and 200 ml of normal heptane was added. The contents were mixed until the mixture was fully dispersed and then boiled under reflux for 1 h. The flask was removed, and the contents were cooled at the end of this period and stored in a dark cupboard for 72 h. The filter paper should be placed in the filter funnel. Next, without agitation, the liquid was decanted into the filter paper, and then the residue in the flask was transferred as completely as possible with successive quantities of hot heptane. The flask used in this step was set aside without washing for use in the next step. The filter paper and contents were removed from the funnel, placed in the reflux extractor, and refluxed with heptane for 1 h.

It should be noted that a different flask from the initial one was used in this stage. Finally, the flask used in the second step was replaced, 100 ml of toluene was added, and refluxing continued until all asphaltene had been dissolved from the paper. Next, the flask's content was transferred to a clean and dry evaporating vessel, weighted by tare against a similar dish, and then toluene was removed by evaporating on a boiling water bath. The procedure was completed by drying the dish and contents in the oven for 30 min and re-weighted by tare against the dish used previously. 

**Figure 1:**
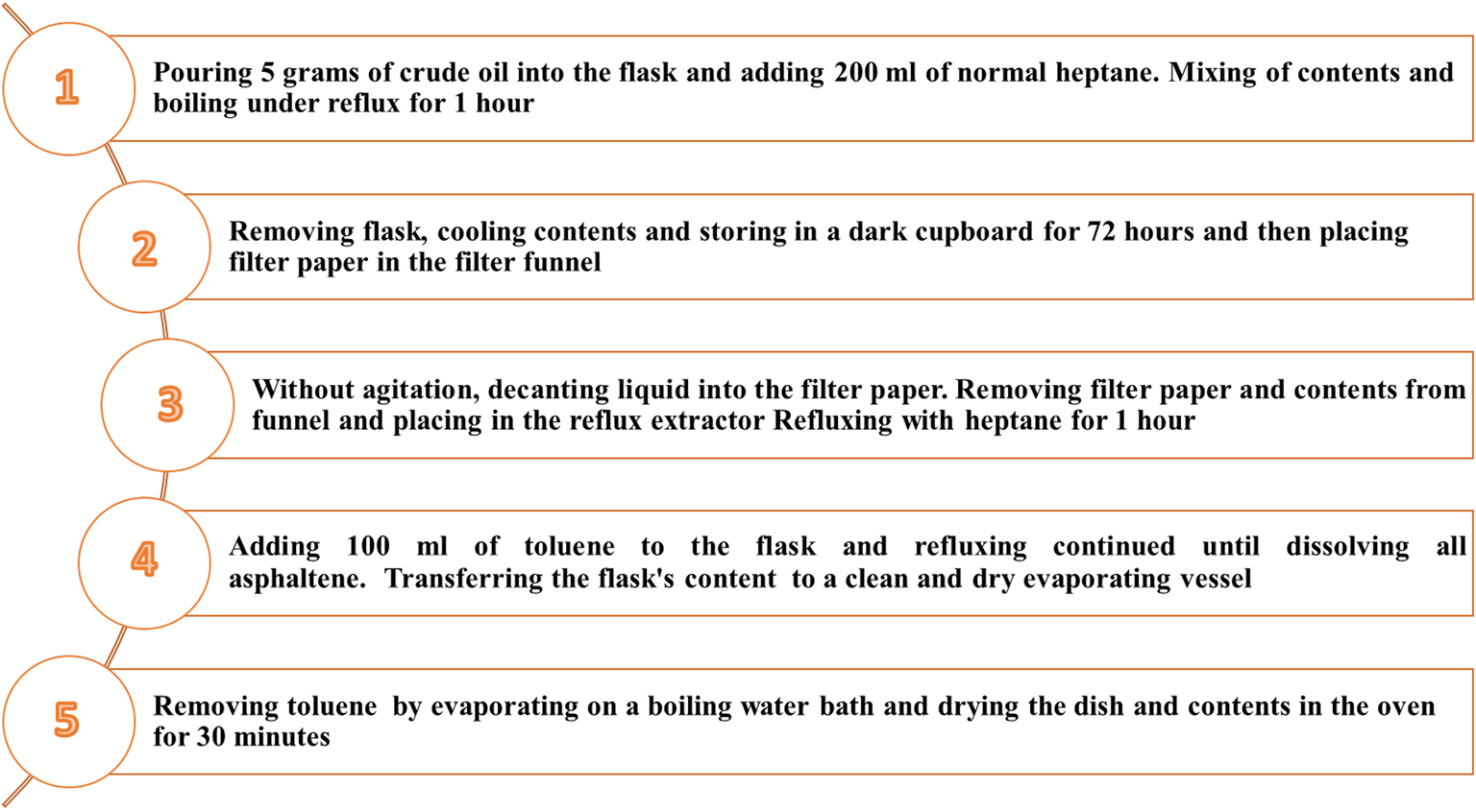
The flow chart for measuring asphaltene contents in crude oil based on the IP-143 method.

Figure 1 shows the summarized procedure for measuring asphaltene contents. The amount of asphaltene content, C, in % (m/m), was evaluated using Eq. (1):

1 $$ C = \frac{100 \times M \times R}{{G \times D}} $$

where M is the mass of asphaltenes (g), R is the mass of residue from distillation (g), G is the mass of the residue aliquot (g), and D is the mass of crude oil sample (g).

### Surface modification of nanoparticles by oleic acid

Surface modification of nanoparticles in porous media is crucial due to its significant impact on transport and reactivity within these environments. Studies have shown that surface modifications enhance the transport of nanoparticles through porous media by increasing colloidal stability, altering surface wettability, and delaying the precipitation behavior of substances like asphaltene^45^. The physicochemical parameters of the solution, nanoparticle surface properties, and flow rate also play a vital role in nanoparticle deposition and mobility in porous media^46^. Surface modification of nanoparticles using oleic acid has been a subject of interest in various studies. Oleic acid, known as a water-fast unsaturated fatty acid, is an effective ligand for stabilizing nanoparticles due to its ability to absorb onto the nanoparticle surface through carboxylate binding^47^.

However, challenges exist in the modification process, such as the limited accessibility of chemical reagents to the double bond in oleic acid due to steric hindrance, complexity in phase-transfer processes, prolonged reaction times, and potential increase in nanoparticle size after ligand exchange^48^. Studies have shown that oleic acid-modified nanoparticles exhibit improved dispersion capabilities, attributed to the reduction of surface energy and dipolar attraction of the nanoparticles^49^. Additionally, the use of oleic acid as a coating material has been proven to yield monodispersed, uniform, and stable nanoparticles, making it a promising modifier for nanoparticle surface functionalization^47^. In the context of EOR applications, it was demonstrated that nanoparticles, when treated with oleic acid, can alter surface wettability from water-wet to oil-wet or vice versa^50^.

Therefore, oleic acid was utilized as a coupling agent for the surface modification of nanoparticles based on the following procedure to determine the onset point of asphaltene precipitation^51,52^. Additionally, heptane was selected as a solvent because it can efficiently dissolve both oleic acid and the nanoparticles, facilitating the mixing process and ensuring uniform distribution of the nanoparticles in the solution. Moreover, heptane's low boiling point allows for easy removal from the solution after the mixing process, leaving behind the desired mixture of oleic acid and nanoparticles for further experimentation. It should be noted that the procedure is the same for both SiO_2_ and CaCO_3_ nano-inhibitors. In this regard, 8 ml of oleic acid was dissolved in 300 ml of heptane and mixed. Subsequently, 10 g of nanoparticles were added to the solution, and at 70 °C, it was stirred for 4 h^51,53^. Then, the solution was filtered, and the precipitate was thoroughly washed with alcohol and distilled water. Finally, the precipitate was stored in a vacuum oven for 24 h to obtain a white powder of nanoparticles modified by oleic acid.

### Nano-inhibitors characterization

The morphology of nano-inhibitors was analyzed by Field Emission Scanning Electron Microscope (FE-SEM3200, KYKY Co., China) with a maximum voltage of about 30 kV. Infrared spectroscopy of the samples was taken by FTIR Spectrometer (L1390021, Perkin Elmer Co., USA).

### Nano-inhibitors stability analysis

The stability of nanofluid was evaluated by two different methods: visual observation and UV Spectrophotometry^54^. In the first technique, nanoparticles were dispersed in distilled water by a probe-type sonicator, and the stability of the solution was examined by direct visual observation at the time steps of 2 h, 4 h, and 24 h after the dispersion of nanoparticles. In the second method, an ultraviolet–visible spectrophotometer (UV-1280, Shimadzu Co., Germany) was used to analyze the optical absorbance of nanoparticles by absorbance time, providing a way to investigate the aggression of nanostructures.

### Measurement of asphaltene onset

A viscosity measurement technique was applied to measure the onset of asphaltene precipitation, and here, normal heptane was chosen as a precipitant. First, several oil samples were prepared and weighed. Then, specific amounts of nano-inhibitors in the concentrations of 0.01, 0.1, and 0.5 wt.% were mixed with oil samples in a closed container. The concentrations of SiO_2_ and CaCO_3_ nano-inhibitors were chosen based on various factors, including prior research, preliminary tests, and economic factors. These concentrations (0.01, 0.1, and 0.5 wt.%) were selected due to their applicability to real-world scenarios and their anticipated efficacy in inhibiting asphaltene precipitation and oil production mechanisms. The mixture was stirred for 2 h at 50 °C to ensure complete dispersion of nano-inhibitors. Next, various volumetric ratios of normal heptane were introduced to the mixture, and final samples were injected into the viscometer to measure the viscosity at atmospheric pressure and temperature of 20 °C.

### Contact angle measurements

**Figure 2:**
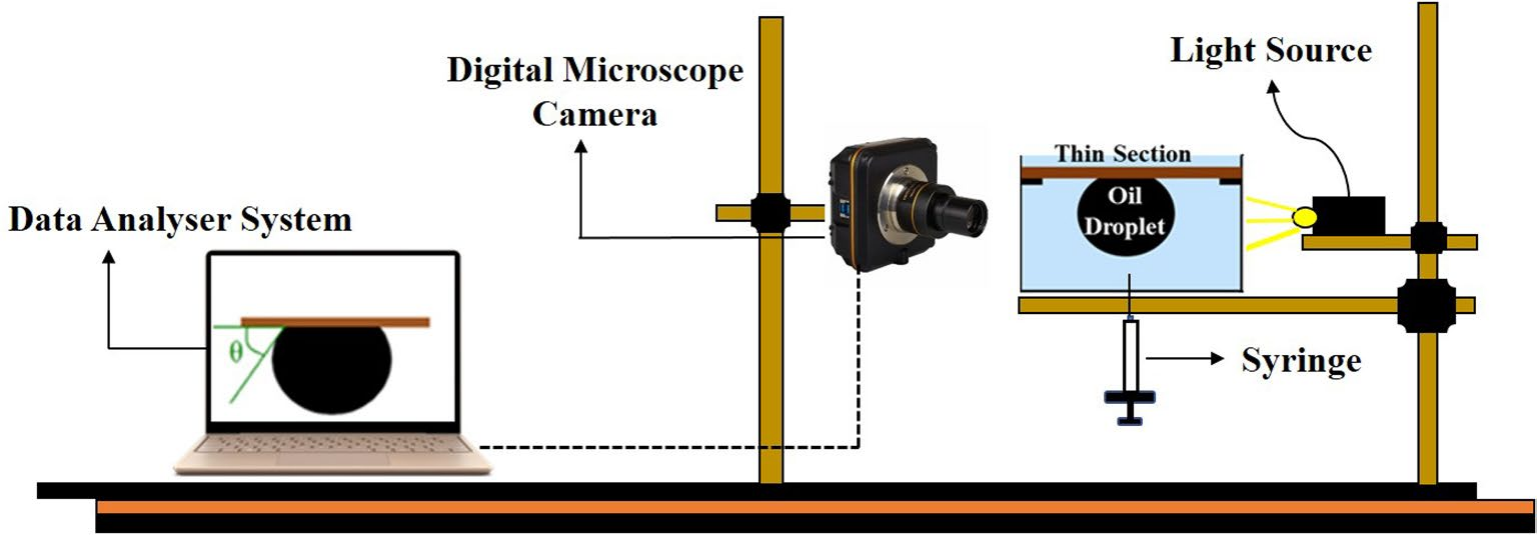
The setup schematic used for contact angle measurements.

The influence of commercial CaCO_3_ and SiO_2_ nanoparticles on wettability alteration of oil-wet glass thin-section was tested by the sessile drop method. As shown in Fig. 2, an oil-wet glass thin-section was placed at the top of the glass container filled with distilled water. Then, an oil drop was injected by a syringe and placed on the bottom of the glass surface. Afterward, the image of an oil drop on the surface of the glass was captured with a high-resolution camera and sent to a computer for future analysis^55^.

### Interfacial tension measurements

The interfacial tension between oil and water was determined using the pendant drop method, following this procedure^56^: Initially, a needle or capillary tube was vertically mounted, and 25 cm^3^ of the prepared nanofluid solution (water phase) was poured into it. Subsequently, a syringe pump was utilized to dispense an oil drop onto the tip of the needle or capillary tube, forming a pendant drop under the influence of gravity. Using a high-resolution camera or microscope, an image of the pendant drop was captured, and its dimensions, such as diameter, were measured. This data is crucial for calculating the interfacial tension. The captured image was then analyzed using appropriate software to determine the interfacial tension. In this approach, IFT was calculated by Eq. (2)^57^:

2 $$ \gamma = \frac{{\Delta \rho gD^{2} }}{H} $$

where $\Delta \rho$ is the density difference between nanofluid and oil (g/cm), g is gravity acceleration (cm/s^2^), D is the largest formed oil drop diameter (cm), and H is the drop shape dependent parameter that is a function of shape factor, S = d/D, where d is horizontal drop oil diameter from distance D above an oil drop.

### Micromodel flooding

A 2-D glass micromodel with a desired pattern (designed by Corel Draw software) was used to observe fluid flow through porous media. To simulate fluid injection conditions similar to real oil reservoirs, a two-dimensional glass micromodel, prepared from thin section images of dolomite rock, was utilized. In this model, fluid injection and crude oil production ports face each other. The characteristics of the designed micromodel are exhibited in Table 5.

**Table 5:** The properties of designed micromodel.

Pattern type	Dimensions (mm)	Depth (µm)	Porosity (%)	Permeability (mD)	Pore volume (cc)	Aspect ratio	Coordination number
Non- homogeneous	60 × 60	60	38	890	0.1	1.6–6	2–6

Most oil reservoirs are initially oil-wet, meaning a thin layer of oil adheres to the inner surface of the pores of the reservoir rock before any flooding process occurs. To replicate real reservoir conditions in the laboratory, glass micromodels must be oil-wet before conducting any injection scenarios. The procedure is detailed below:

First, a NaOH solution was used to saturate the micromodel for 1 h^58–60^. Then, to remove any impurities present in the pore throat of the porous media, the micromodel was washed with DI water and dried at 200 °C for 20 min. In the next stage, a solution consisting of 98% pure toluene and 2% trichloromethylsilane was injected into the micromodel and saturated for at least 5 min. Finally, the micromodel was washed with methanol and then dried in the oven for 30 min^61^.

**Figure 3:**
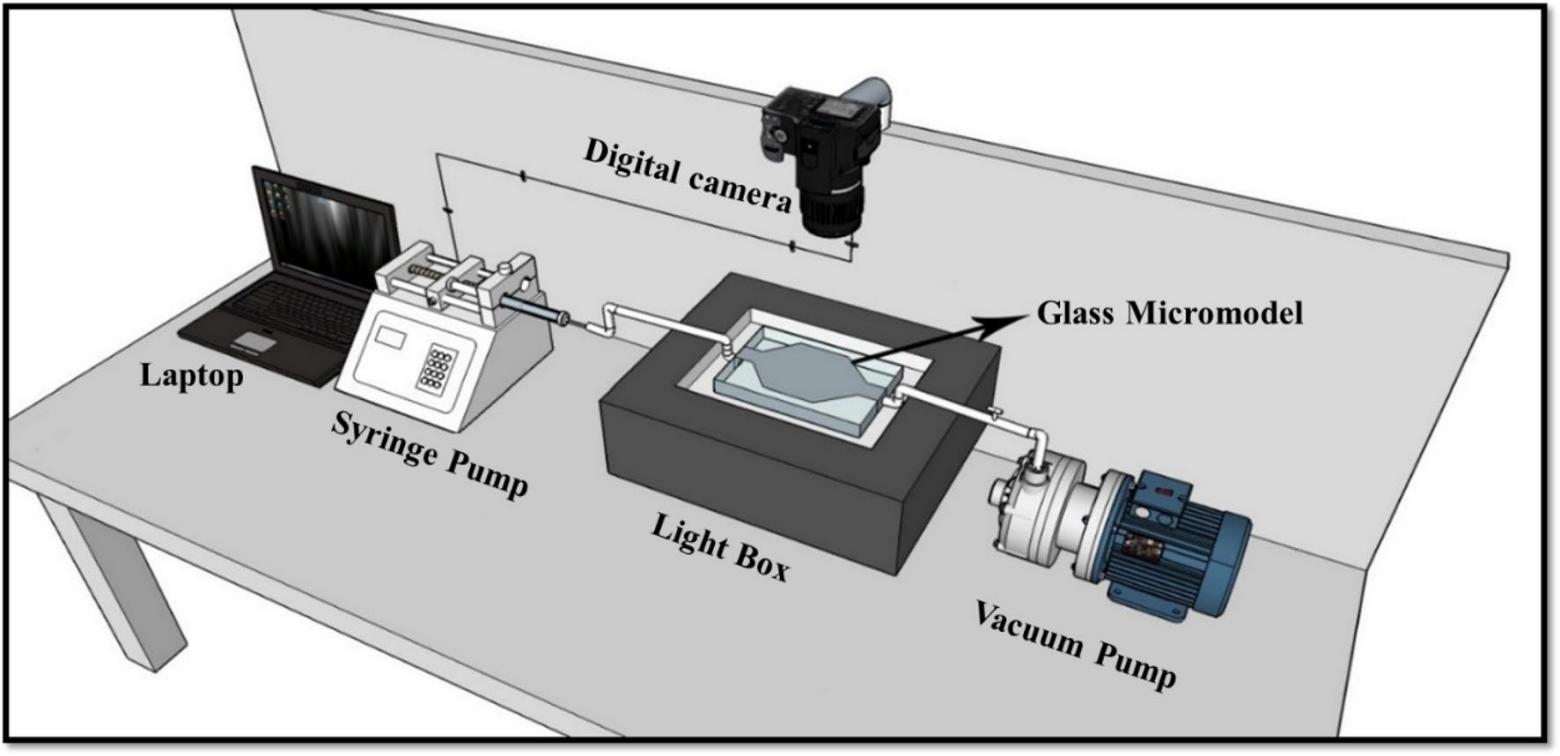
The schematic of micromodel flooding setup.

Afterward, crude oil was injected into the micromodel at a rate of 1 ml/h. Subsequently, 4 PV of nanofluid solution was injected into the system at a flow rate of 0.05 ml/h. Throughout this procedure, a high-resolution camera captured photos and videos of the process. The photos of the porous medium at different injection times were then analyzed using computer image processing software, such as Photoshop. The counting of the number of black pixels (representing the remaining oil in the porous medium) before and after flooding forms the basis of this method for determining the oil recovery factor^13^. Figure 3 illustrates the micromodel flooding setup for evaluating oil recovery efficiency.

## Result and discussion

### Nano-inhibitors characterization

**Figure 4:**
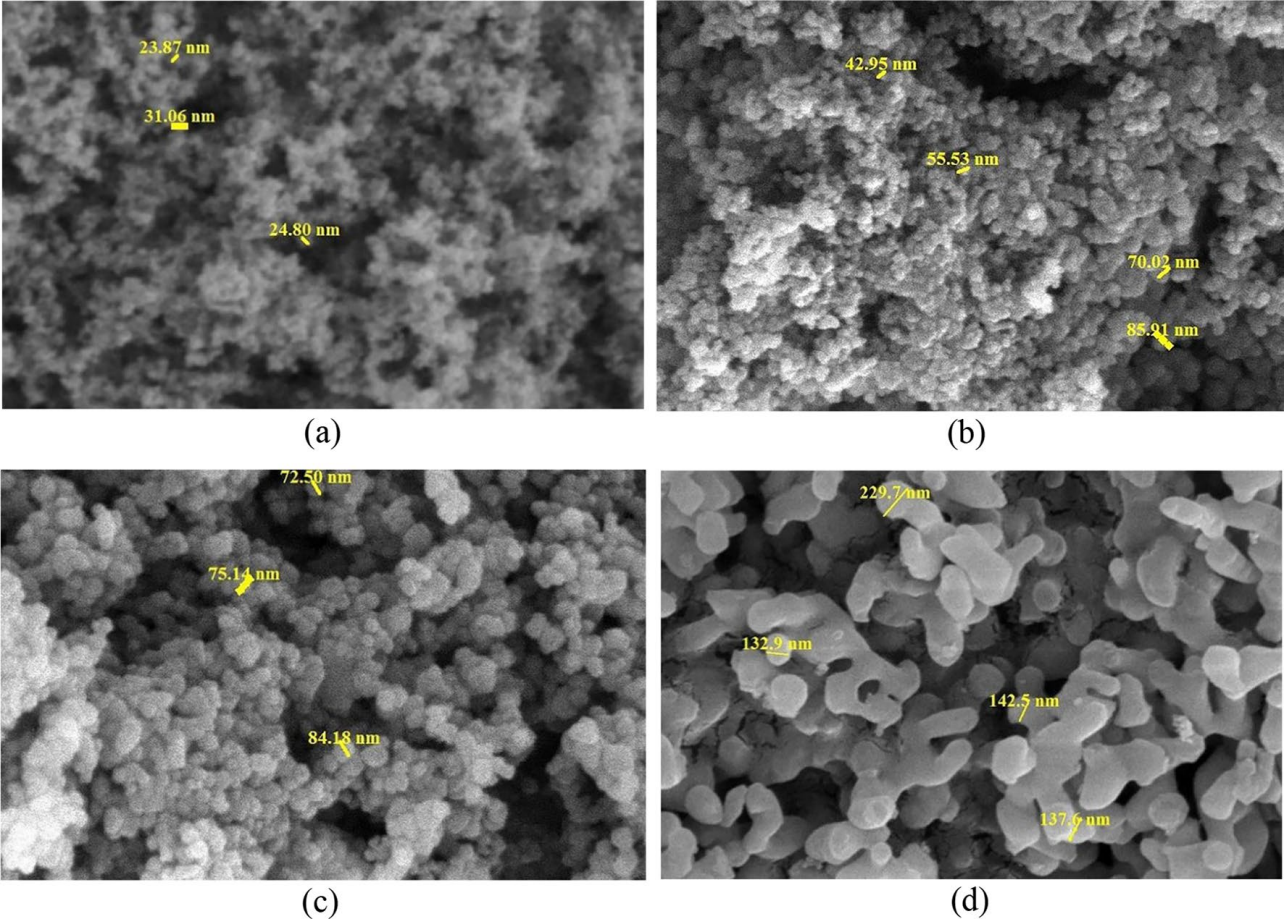
FE-SEM images of nanoparticles structure: (**a**) uncoated SiO_2_ nanoparticles, (**b**) SiO_2_ nanoparticles coated by oleic acid, (**c**) uncoated CaCO_3_ nanoparticles, (**d**) CaCO_3_ nanoparticles coated by oleic acid.

FE-SEM images of SiO_2_ and CaCO_3_ nano-inhibitors before and after surface modification are shown in Fig. 4a–d. According to FE-SEM images, the spherical morphology of utilized nanoparticles can be confirmed. SiO_2_ nano-inhibitor has a smaller size and lower weight than CaCO_3_ nano-inhibitor, which has increased the specific surface area of SiO_2_ nano-inhibitor. Moreover, the low weight of SiO_2_ causes much more consumption of this nano-inhibitor.

The size of SiO_2_ and CaCO_3_ nano-inhibitors before and after surface modification is illustrated in Table 6. Note that after surface modification, the size of nano-inhibitor particles increases due to the coating of oleic acid. It should be noted that all FE-SEM analyses were conducted on diluted nanofluid samples (consisting of SiO_2_ and CaCO_3_ nanoparticles dispersed in water) to enhance image quality and minimize the presence of agglomerated particles in the captured images. However, achieving complete separation of the particles in this analysis is not feasible. Subsequently, Image J software was utilized to determine the size dispersion of the synthesized particles. In this software, a large number of particles were analyzed to measure the nanoparticle size, and the final size was reported based on the average of these measurements. Consequently, it can be asserted that the reported size for each nanoparticle is very close to the actual size.

**Table 6:** The size of SiO_2_ and CaCO_3_ nanoparticles before and after surface modification.

Type of nano-inhibitor	Before surface modification (nm)	After surface modification (nm)
SiO_2_	25.3 ± 2.2	63.6 ± 1.8
CaCO_3_	77.2 ± 2.6	160.4 ± 3.1

**Figure 5:**
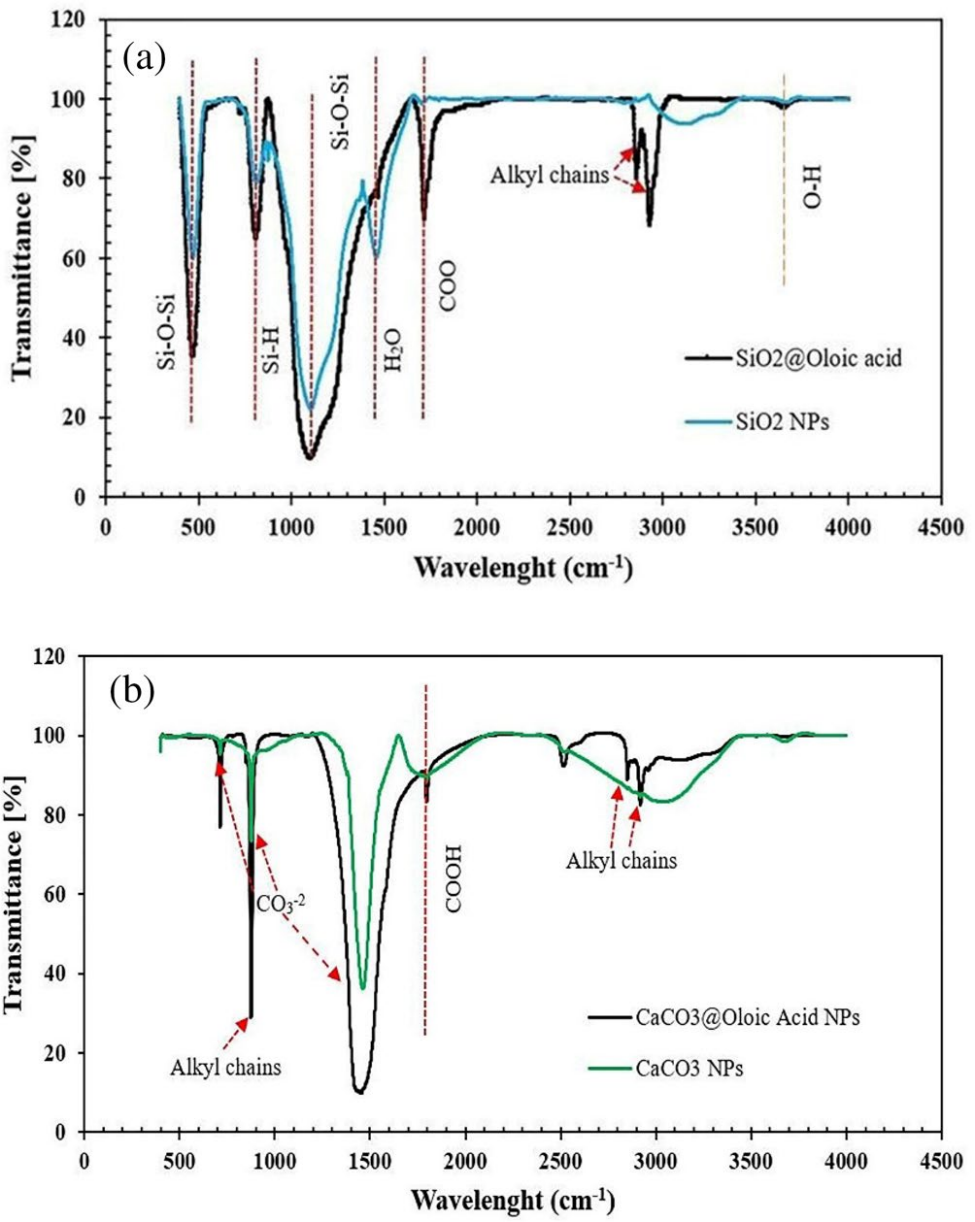
FTIR analysis of (**a**) coated and uncoated SiO_2_, (**b**) coated and uncoated CaCO_3_ nanoparticles.

Fourier Transform Infrared Spectroscopy (FTIR) analysis investigated molecular bonds in nanostructures. Based on Fig. 5, in the case of uncoated (commercial) SiO_2_ nanoparticles, peaks at 460 and 1104 cm^−1^ belonged to Si–O–Si stretching vibration bonding. Moreover, peaks at 874 and 1480 cm^−1^ were related to Si–H and H_2_O groups respectively, but for modified SiO_2_ nanoparticles, the peaks located around 2862 and 2927 cm^−1^ indicated long alkyl chains present at their surface. Furthermore, the peak at 3750 cm^−1^ was associated to the O–H bond and the peak at 1716 cm^−1^ belonged to the –COOH vibration peak corresponding to oleic acid. In the case of uncoated CaCO_3_ nanoparticles, peaks at 876 and 1472 cm^−1^ show CO_3_^2−^ bending vibration of the calcite polymorph, and the peaks at 715 cm^−1^ indicate CO_3_^2−^ bending vibration of vaterite polymorph. For modified CaCO_3_ nanoparticles, the peak at 1670 cm^−1^ was related to carboxylic groups, confirming the attachment of oleic Acid on the surface of CaCO_3_ nanoparticles. Moreover, peaks at 2861 cm^−1^ and 2934 cm^−1^ were assigned to the long alkyl chains of oleic acid.

### Nanofluid stability analysis

**Figure 6:**
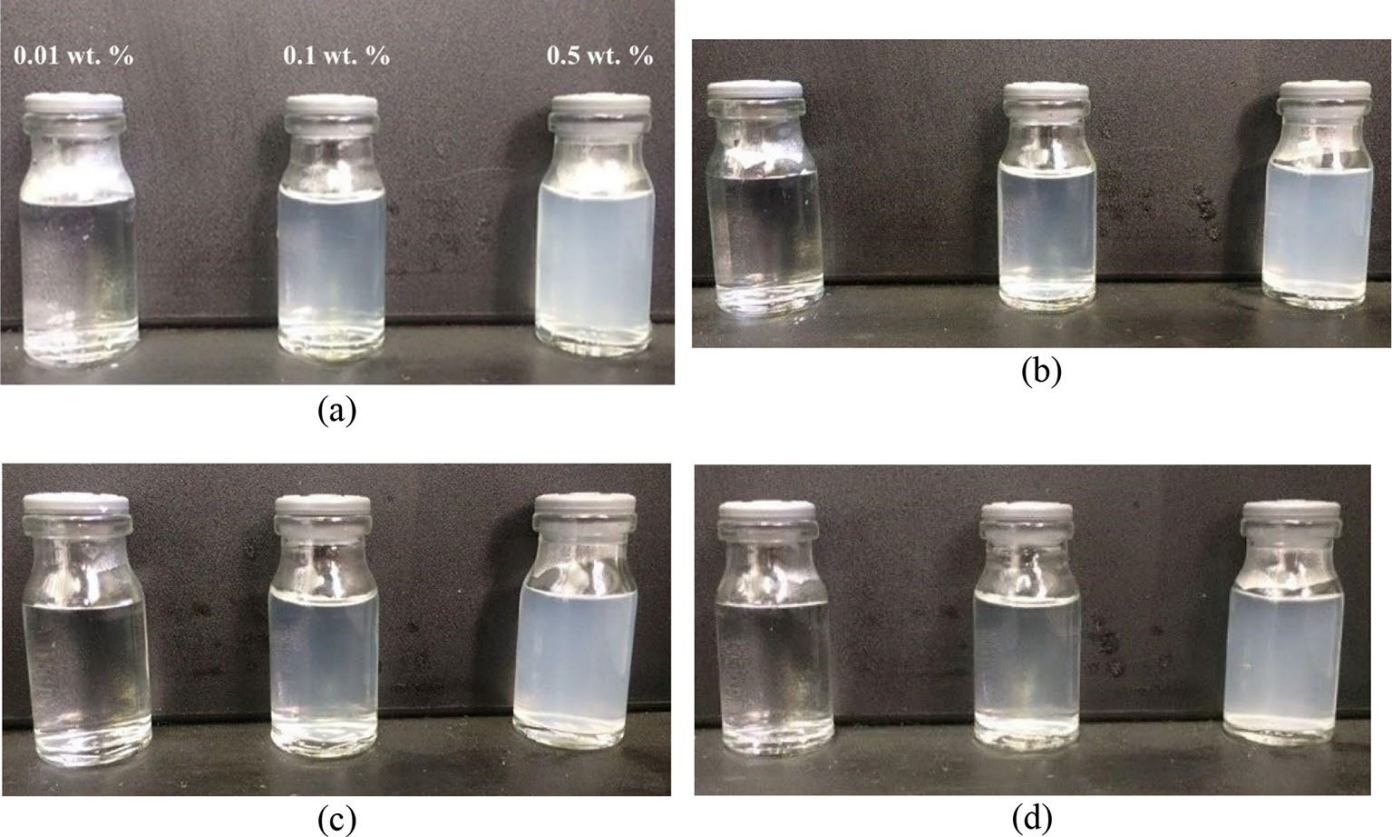
Visual observation analysis for uncoated SiO_2_ nanoparticles (**a**) initial time, (**b**) after 2 h, (**c**) after 4 h, (**d**) after 24 h.

**Figure 7:**
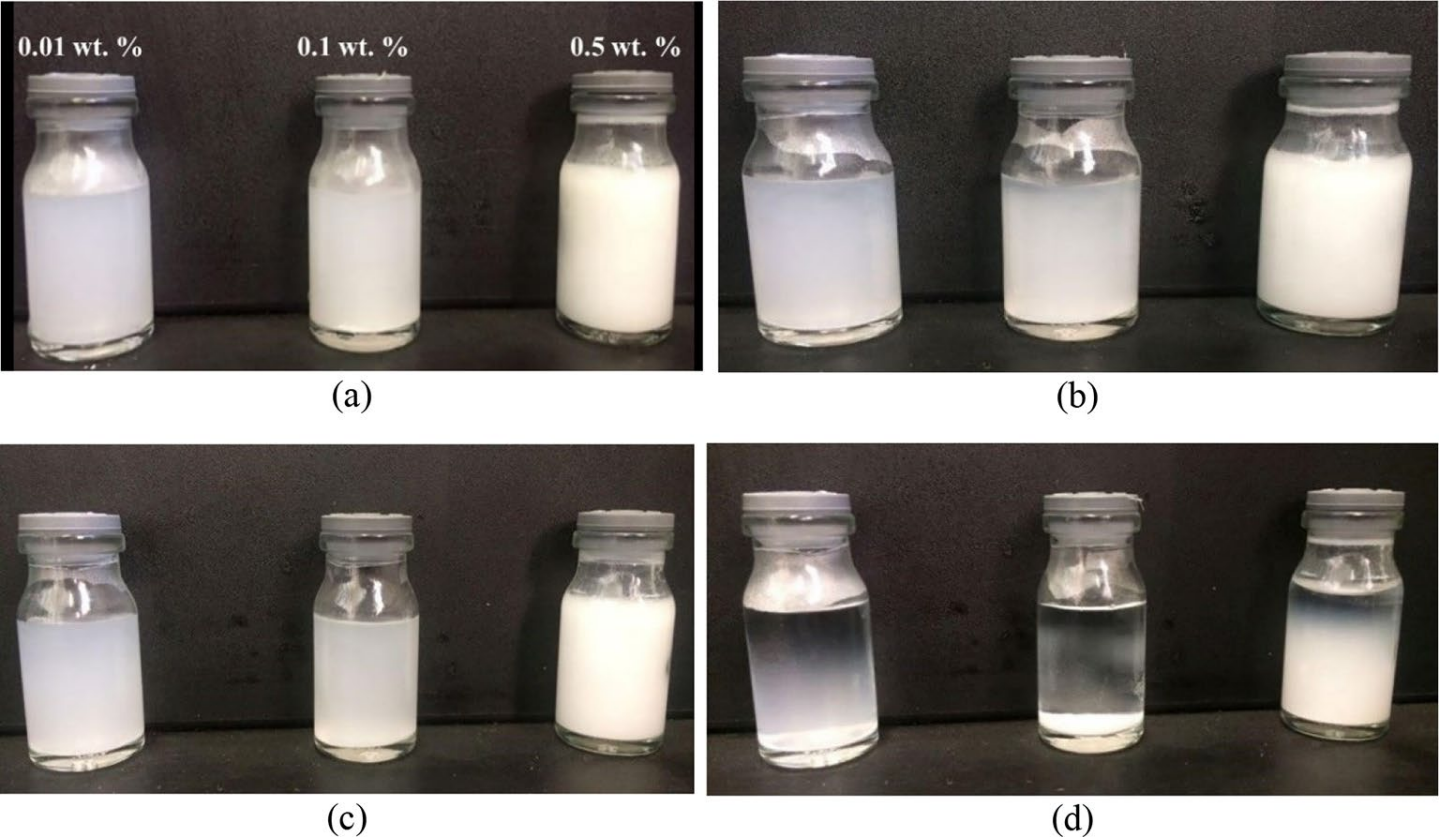
Visual observation analysis for uncoated CaCO_3_ nanoparticles (**a**) initial time, (**b**) after 2 h, (**c**) after 4 h, (**d**) after 24 h.

Direct visual observation method and UV–Vis Spectrophotometer device were applied to elucidate the stability of nanoparticles. Visual observation was performed for three concentrations (0.01, 0.1, 0.5 wt.%). The results are shown in Figs. 6 and 7. Based on the obtained results, utilized nanoparticles exhibited high stability, particularly in the first 4 h. The time of 4 h is vital because micromodel tests last about 3–4 h; during this time, nanofluids should be stable and not settle.

**Figure 8:**
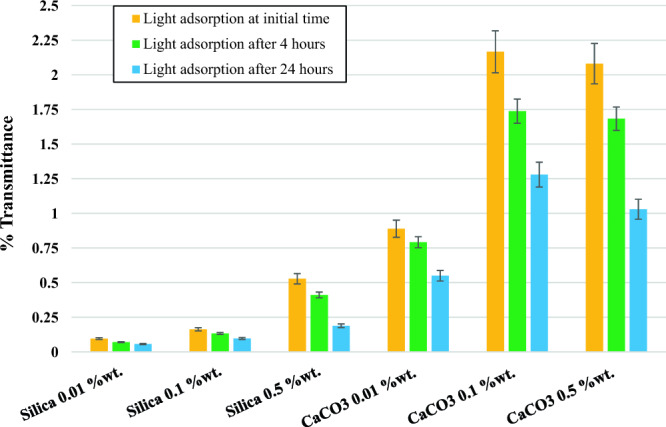
The amount of light adsorption by different concentrations of nanofluids.

Figure 8 summarizes the results of the UV–Vis spectrophotometer at the initial time and after 4 h. This method is based on the absorption of light by nanofluids. The results of the UV–vis analysis after 4 h showed no significant change compared to the initial results obtained immediately after preparing the solution. As shown in this figure, after 4 h, the amount of light absorbed by the nanofluid solution decreases due to the precipitation of nanoparticles. However, this reduction in light adsorption over 4 h is small and averages less than 5% for almost all samples, suggesting that the nanoparticles are stable in the fluid over 4 h. However, after 24 h, the light absorption by the nanofluid solution decreases significantly, indicating a loss of stability, rendering it unsuitable for injection processes. Therefore, developing an optimal method for surface modification of these nanoparticles is crucial for their effective use in real operations.

### Determining the onset point of asphaltene precipitation

**Figure 9:**
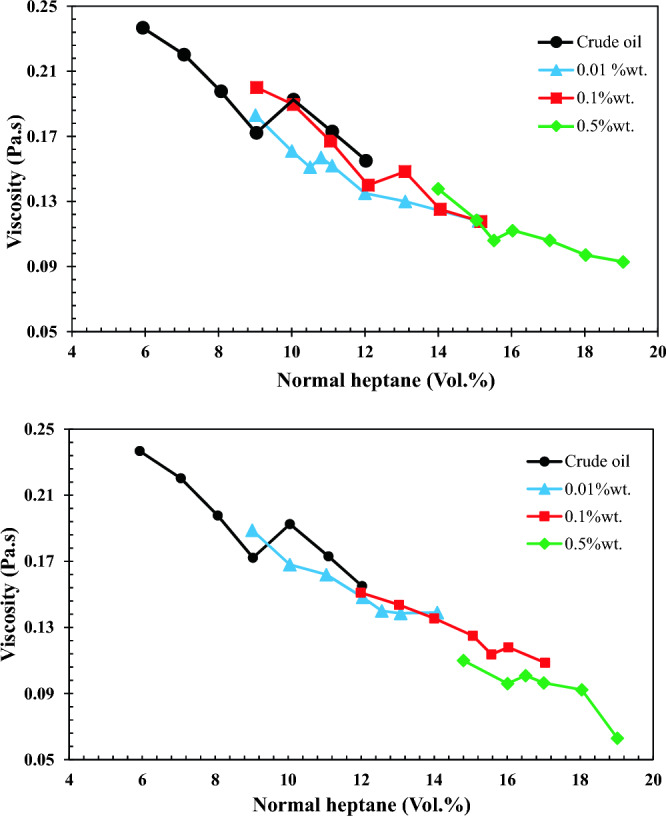
Changes in viscosity of (**a**) SiO_2_ and (**b**) CaCO_3_ nanofluid at different concentrations versus volume percentage of normal heptane.

According to the results of the IP-143 test, the amount of asphaltene in the crude oil was calculated at 26%. Figure 9 elucidates the results of the viscometry method to determine the onset point of asphaltene precipitation. Initially, the onset of crude oil was measured without the presence of nano-inhibitors as an index for comparison of nano-inhibitors' efficiency in preventing the formation of asphaltene precipitation. As shown in Fig. 9, the viscosity of crude oil (non-precipitating solvent) continuously decreases with increasing normal heptane (precipitant solvent) concentration until it reaches a deviation point. This point is called the onset of asphaltene precipitation. The onset of asphaltene precipitation from 10 vol. % of normal heptane for crude oil increased to 10.75, 13, and 16 vol. % of normal heptane for 0.01, 0.1, and 0.5 wt.% of SiO_2_ nano-inhibitors, respectively which indicates the positive performance of nano-inhibitors in preventing the formation of asphaltene precipitation. A similar trend was observed in the case of CaCO_3_ nanoparticles.

The performance mechanism of nano-inhibitors depends on forming hydrogen bonding with an activated site on the surface of asphaltene molecules. In other words, due to polar interaction, nano-inhibitors attach to the asphaltene molecules and make them suspended in crude oil. More polar interaction results in stronger hydrogen bonding, which postpones the onset of asphaltene precipitation. As discussed earlier, both nano-inhibitors were coated with oleic acid to be oil-wet. The FTIR spectroscopy results in Fig. 5 showed that the carboxylic acid functional group attached to the surface of nanoparticles. Therefore, it can be inferred that they have acidic chemical nature. According to Nassar et al. works^62^, nanoparticles with acidic chemical nature perform better in adsorbing asphaltene molecules than amphoteric nanoparticles due to stronger polar interaction. It is because of the type of constitutive acid in their structure. Constitutive acid is divided into Bronsted and Lewis acids^63^.

**Figure 10:**
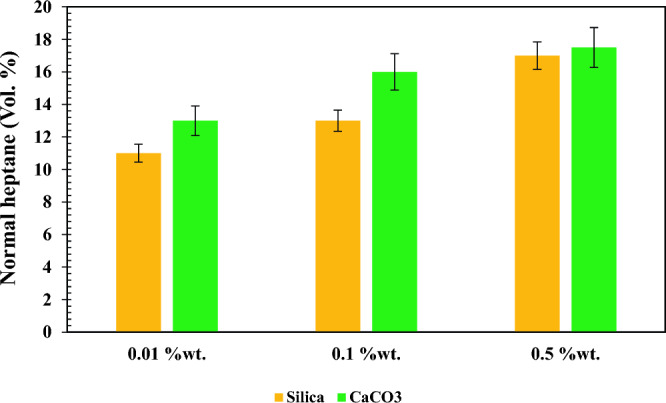
Comparing the performance of SiO_2_ and CaCO_3_ nano-inhibitors on the onset point of asphaltene precipitation.

Bronsted acids are compounds that can donate a proton (H^+^), whereas Lewis acids are electron pair acceptors. Bronsted acid sites can form a stronger bond than Lewis acid^64^. Thus, it can be concluded that both nano-inhibitors are formed mainly from the Bronsted acid site, and their hydrogen bonding is strong enough to delay the formation of asphaltene precipitation. However, the performance of CaCO_3_ and SiO_2_ nano-inhibitors are not similar despite this fact. For more comparison, the influence of SiO_2_ and CaCO_3_ nano-inhibitors on the onset point of asphaltene precipitation is depicted in Fig. 10. According to this figure, CaCO_3_ nano-inhibitor exhibited better performance in low concentrations compared to SiO_2_ but in higher concentrations, it did not show much superiority over SiO_2_ nano-inhibitor.

For better understanding, in Table 7, the index for evaluating the performance of nano-inhibitors in low and high concentrations was assigned to the changes in the volume percentage of heptane. In this point of view, it can be said that at low concentrations, SiO_2_ and CaCO_3_ nanoparticles increased onset points to 17.31% and 18.75%, respectively, which exhibit higher efficiency of CaCO_3_ nanoparticles. However, SiO_2_ nano-inhibitor acted better than CaCO_3_ at high concentrations. This issue becomes significant when economic conditions should be considered in the large-scale application of nano-inhibitors. The reason for these observations is that in high concentrations of CaCO_3_ nano-inhibitor, its solubility in water decreases^65^. Accordingly, the solubility of CaCO_3_ nano-inhibitor in oil reduces at higher concentrations. On the other hand, with an increasing concentration of SiO_2_ nano-inhibitors, its solubility in water increases, and as a result, appreciable changes can be seen in the onset of asphaltene precipitation.

**Table 7:** The onset of asphaltene precipitation based on volume% of normal heptane.

Nano-inhibitor	Concentration (wt.%)			Value of changes (%)	
	0.01	0.1	0.5	(From 0.01 to 0.1 wt.%)	(From 0.1 to 0.5 wt.%)
SiO_2_	10.75	13	16	17.31	18.75
CaCO_3_	13	16	16.5	18.75	3.04

### Wettability measurements

**Figure 11:**
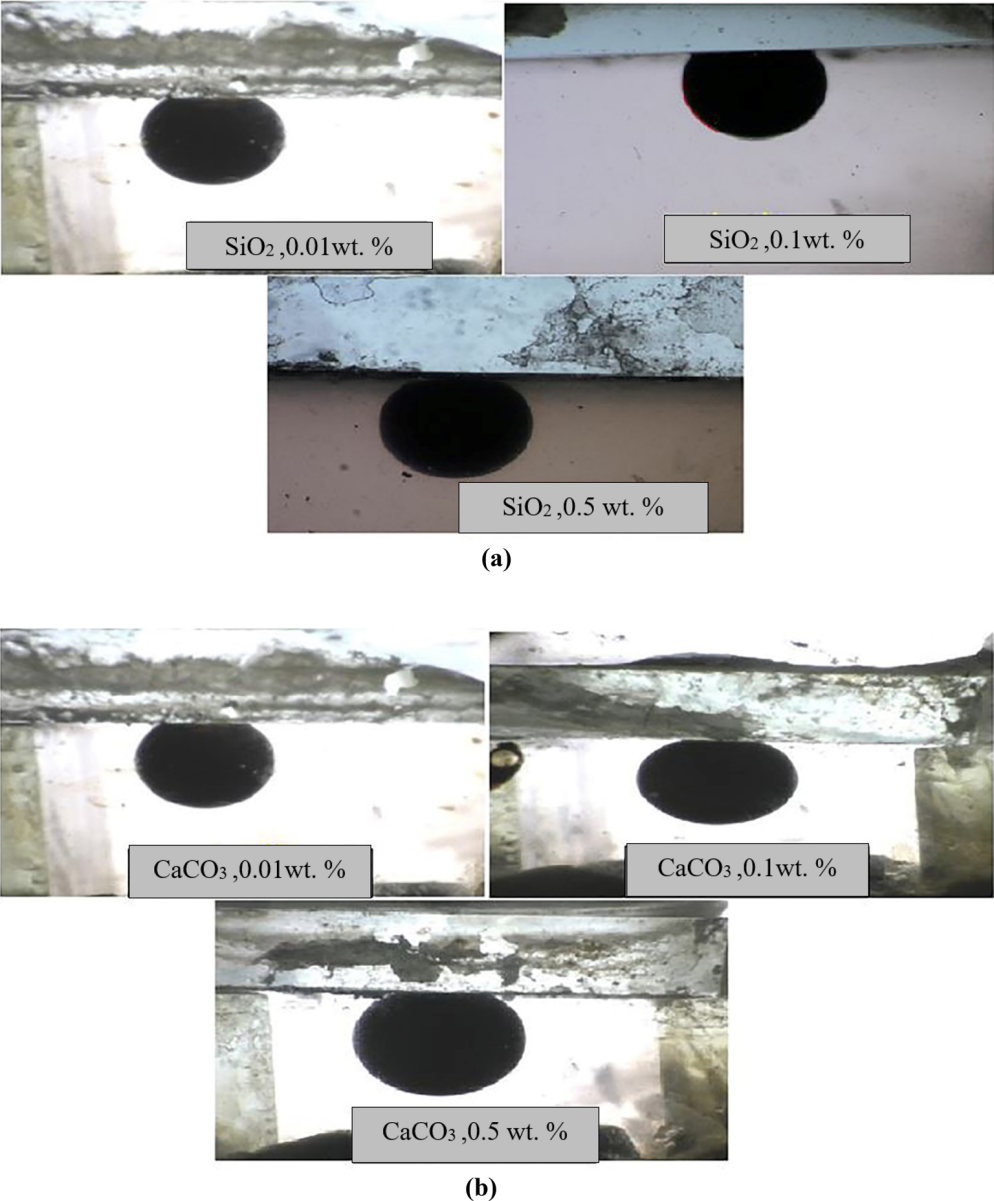
The results of contact angle measurements based on (**a**) SiO_2_ nano inhibitors, (**b**) CaCO_3_ nano inhibitors.

**Figure 12:**
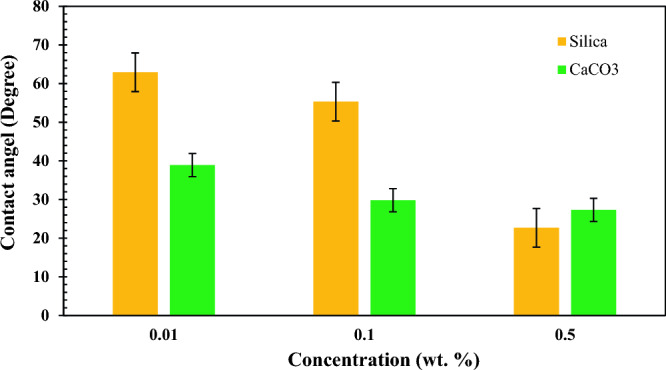
Changes of contact angle at different concentrations of nano-inhibitors.

Before conducting contact angle tests, the contact angle between the water droplet and the glass thin-section was measured with the value of 143.5°, confirming that the surface of the thin-section was strongly oil-wet. In Fig. 11a,b, the results of contact angle measurements are depicted. Figure 12 shows the changes in contact angles versus different concentrations of nano-inhibitors. Here, the lower contact angle indicates the higher tendency of the glass surface toward water-wet.

In low concentrations of nano-inhibitors, CaCO_3_ nanoparticles had a lower contact angle than SiO_2_ nanoparticles. It means that the glass surface becomes more water-wet than in the case of using SiO_2_ nanoparticles. But in higher concentrations, SiO_2_ nanoparticles perform better for changing glass wettability toward water-wet. Polar interactions among nano-inhibitors and crude oil should be considered to interpret the reason for this observation. Weak acidic polar silanol functional groups (Si–OH) on the silica surface act as sites for adsorbing polar components in crude oil. Polar bondings between SiO_2_ nano-inhibitor and crude oil molecules are stronger than CaCO_3_ nano-inhibitors because of more positive charges on the surface of SiO_2_ nanoparticles. Thus, the SiO_2_ nano-inhibitor changes the wettability of oil-wet glasses to water-wet more strongly, and the contact angles measured have a lower value. Beyond this, although the fraction of atoms in the bulk of nanoparticles increases with an increase in particle diameter, the fraction of atoms on the surface of nanoparticles decreases. It is clear that atoms at the surface are responsible for interaction, and because breaking bonds costs energy, surface atoms always have higher energy than atoms in the bulk. Therefore, it can be concluded that nanoparticles with lower particle sizes have higher surface energy, resulting in stronger bonding. Here, due to the results of SEM analysis in Fig. 4, SiO_2_ nanoparticles have a lower particle size (higher surface energy), which causes the formation of stronger polar binding with crude oil molecules present on the surface of oil-wet glasses. Thus, they acted better than CaCO_3_ nano-inhibitors for changing wettability toward water-wet.

### IFT measurements

**Figure 13:**
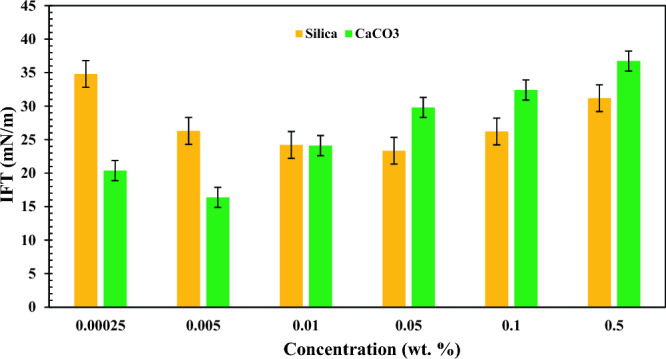
Logarithmic changes of IFT between oil and water versus concentration.

IFT measurements were performed for CaCO_3_ and SiO_2_ nano-inhibitors at different concentrations of 0.00025, 0.005, 0.01, 0.05, 0.1, and 0.5 wt.%, and their results are shown in Fig. 13.

Based on the thermodynamic view, interfacial tension can be explained by changes in Gibbs free energy concerning interfacial area according to Eq. (3):

3 $$ \gamma = \frac{\partial G}{{\partial A}} $$

Although we know that Gibbs free energy is a function of entropy, thus any changes in entropy can alter interfacial tension. At first, when there are no nanoparticles at the water/oil interface, water molecules at the interface are pretty small, and the entropy is at a high level. When nanoparticles with larger particle sizes are present in the aqueous phase, nanoparticles are placed at the interface of water/oil and displace water molecules. It is due to molecular interaction between Si^4+^ and Ca^2+^ ions and oxygen molecules of the carboxylic groups, polar components in crude oil. Therefore, the entropy value decreases, resulting in reduced interfacial tension. The trend of IFT reduction continues until reaching an optimum value which IFT has the lowest available value. The optimum concentration for SiO_2_ and CaCO_3_ nano-inhibitors occurred at 0.05 and 0.005 wt.%, respectively. With increased nanoparticle concentration beyond the optimum, nanoparticles become tightly packed, increasing intermolecular forces per unit area. This increase in the force per unit area induces a resistance toward the interface layer’s deformation, and interfacial tension increases.

At low concentrations, the performance of CaCO_3_ nanoparticles is better than SiO_2_ nanoparticles for IFT reduction. It can be explained by the results of molecular dynamic calculations by Vollath et al.^66^. They concluded that increasing the nanoparticle's size decreases the value of surface free energy. In Table 6, it is demonstrated that the size of CaCO_3_ nanoparticles is more than SiO_2_ nanoparticles, which shows that the surface free energy of CaCO_3_ nanoparticles is lower than SiO_2_ nanoparticles. Lower surface free energy results in weaker molecular interaction at the interface of two phases which causes IFT reduction. However, in higher concentrations, the effect of nanoparticles' solubility in the aqueous phase is a dominant parameter, and due to the lower solubility of CaCO_3_ nanoparticles, which results in a higher density difference between oil nanofluid solutions, the IFT of water/oil in the presence of CaCO_3_ nanoparticles had higher value compared with SiO_2_ nanoparticles.

### Flooding experiments

**Figure 14:**
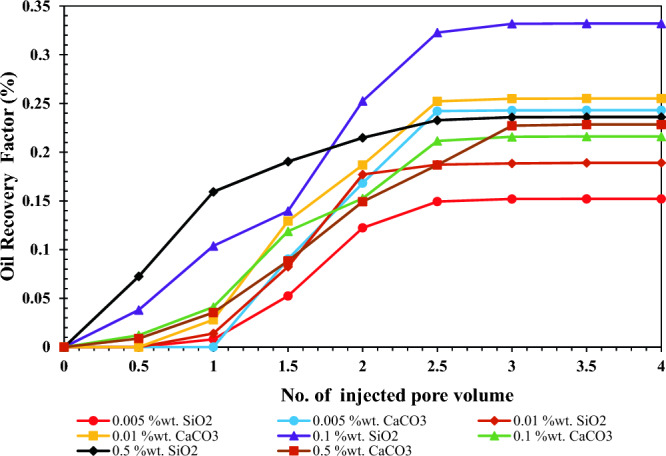
Oil recovery efficiency versus number of PV of injected fluid for different concentrations of nanofluids.

For investigating the impact of SiO_2_ and CaCO_3_ nanoparticles on oil recovery efficiency, four different concentrations of nanofluids, including 0.005, 0.01, 0.1, and 0.5 wt.%, were injected into the oil-wet glass micromodel and image analysis was applied for calculation of oil recovery. Figure 14 shows the results of oil recovery versus injected pore volumes. As can be seen, increasing the concentration of SiO_2_ nanoparticles from 0.01 to 0.1 wt.% resulted in the recovery increasing from 18 to 33%, the highest amount of achieved oil recovery among all tests. However, by increasing concentration up to 0.5 wt.%, oil recovery reduction occurred up to 23%, and among the various concentrations of CaCO_3_, more oil was recovered in the case of 0.01 wt.%. The capillary number should be calculated in each case to analyze oil recovery factor results accurately. Capillary number characterizes the ratio of the viscous forces to the surface or interfacial tension forces. It comprehensively describes the simultaneous effect of fluid viscosity, velocity, and interfacial tension, which play remarkable roles in the EOR process. This dimensionless number is defined as^67^:

4 $$ N_{c} = \frac{\nu \mu }{{\sigma \cos \theta }} $$

where ν, μ, σ, and θ are velocity, viscosity, interfacial tension, and contact angle, respectively. Here velocity is the same for all cases and is considered constant. Moreover, the Einstein formula is applied to calculate nanofluid viscosity for dilute suspensions containing spherical particles^68^.

5 $$ \mu_{nf} /\mu_{bf} = 1 + 2.5\varphi $$

where μ_nf_, μ_bf,_ and φ are nanofluid viscosity, base fluid viscosity (DI water), and particle volume concentration. φ is calculated by Eq. (6), and the capillary number is presented in Table 8.

6 $$ \varphi \times 100 = \frac{{\left[ {W_{particle} /\rho_{particle} } \right]}}{{\left[ {W_{particle} /\rho_{particle} } \right] + \left[ {W_{fluid} /\rho_{fluid} } \right]}} $$

**Table 8:** Calculated capillary number.

Nanoparticle	Concentration (wt.%)	*φ*	Viscosity	*σ*	*θ*	*N*_*c*_
SiO_2_	0.005	0.0018	1.0047	26.3	72	0.0395
	0.01	0.0037	1.0094	24.21	69.2	0.4184
	0.1	0.0365	1.0912	26.21	55.31	0.1276
	0.5	0.1862	1.4655	31.18	22.71	0.0624
CaCO_3_	0.005	0.0017	1.0042	16.39	48	0.957
	0.01	0.0034	1.0085	24.11	38.92	0.122
	0.1	0.0329	1.0822	32.41	29.18	0.0541
	0.5	0.145	1.3625	36.73	27.31	0.065

Based on the data in Table 8, by increasing the concentration of SiO_2_ nanoparticles, the capillary number increases and reaches its maximum value at a concentration of 0.1 wt.%, and after that, reduction occurs. When the capillary number is high, two factors increase the oil recovery. The first factor is the higher viscosity of the injected fluid, which causes more oil to be pushed, and sweeping efficiency increases. The second factor is the low level of surface tension, as well as wettability alteration in which the tendency of the oil to adhere to the rock surface due to the presence of nanoparticles is low, and oil is more easily separated from the rock surface. These two factors cause the oil recovery to be higher in higher capillary numbers. The same condition occurs in the case of CaCO_3_ nano-inhibitors, and at an optimum concentration of 0.01 wt.% (maximum capillary number), oil recovery increases up to 25%. In both cases, when the concentration of injected nanofluid exceeds the optimum concentration, the amount of interfacial tension increases on a microscopic scale. On the other hand, nanoparticle agglomeration occurs on a macroscopic scale, which plugs the pores of the porous medium. It is more severe in CaCO_3_ nanoparticles because they have larger particle sizes than SiO_2_ nanoparticles and are more likely to pore plugging. Therefore, a high concentration of CaCO_3_ has mainly low oil recovery. The lowest oil recovery was observed at a concentration of 0.005 wt.%. In low concentrations of SiO_2_ nanoparticles, the injected fluid has a low viscosity and weak ability to reduce the interfacial tension.

**Figure 15:**
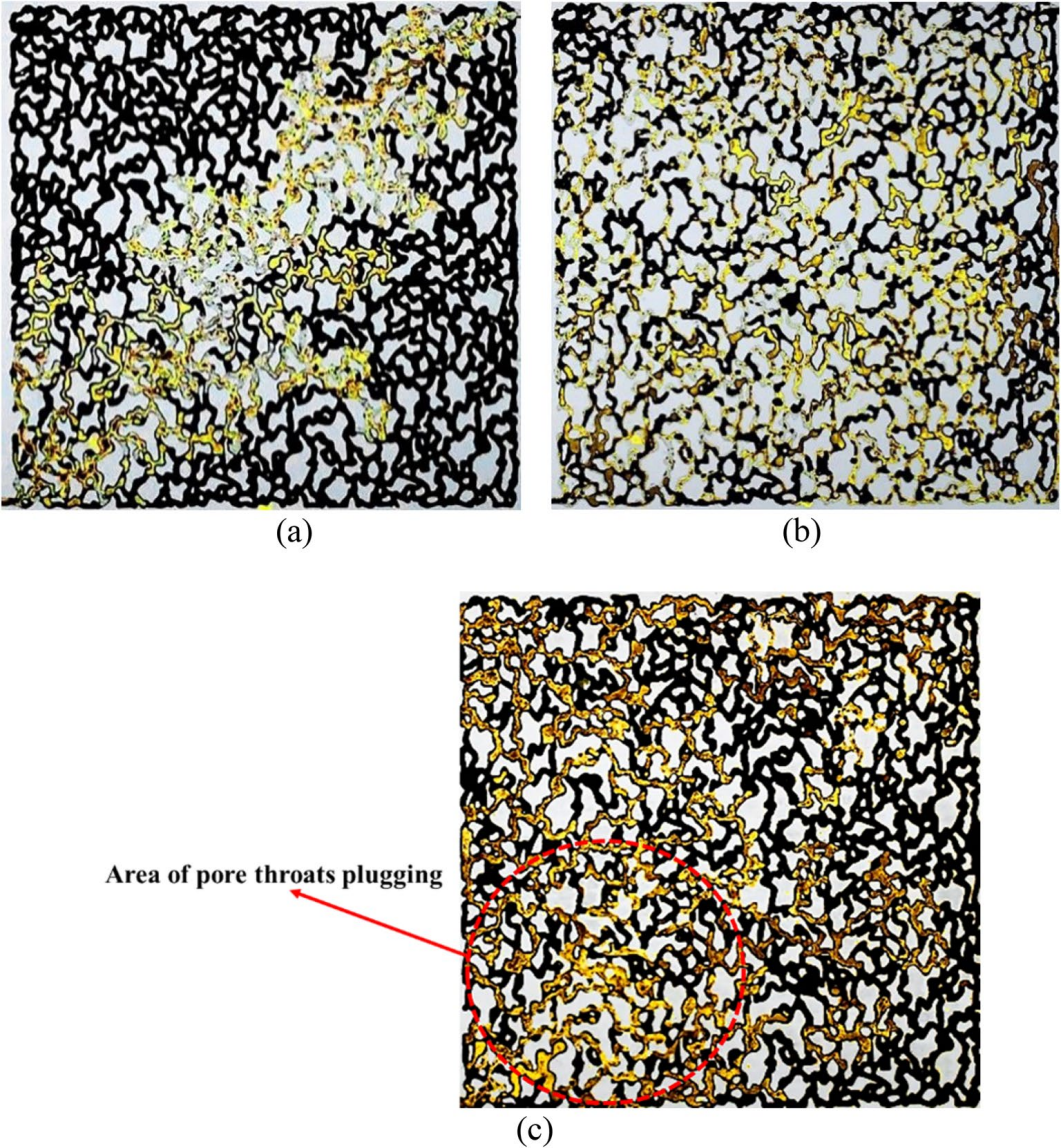
Microscopic images of oil displacement by different concentrations of SiO_2_ nanoparticles (**a**) 0.01 wt.%, (**b**) 0.1 wt.%, (**c**) 0.5 wt.%.

Figure 15 shows microscopic images of nanoparticle flooding at various nanoparticle concentrations. Based on macroscopic images, as SiO_2_ nanoparticle concentration increases from 0.1 to 0.1 wt.%, the fluid front displays a greater tendency towards dispersion, and the ability of fluid to overcome capillary pressure also increases. Additionally, the fingering phenomenon has decreased, and sweep efficiency has improved. On the contrary, as SiO_2_ nanoparticle concentration increases in solution from 0.1 to 0.5 wt.%, due to IFT increasing and also nanoparticles agglomeration, in the area near the inlet of micromodel which is marked in the figure, the throats of the porous medium was plugged by nanofluid solution. As a result, contrary to what was imagined that oil recovery increases with increasing concentration, experiments have shown that this factor will not continuously improve oil recovery, and considering nanoparticle concentration is in high priority before the injection process.

**Figure 16:**
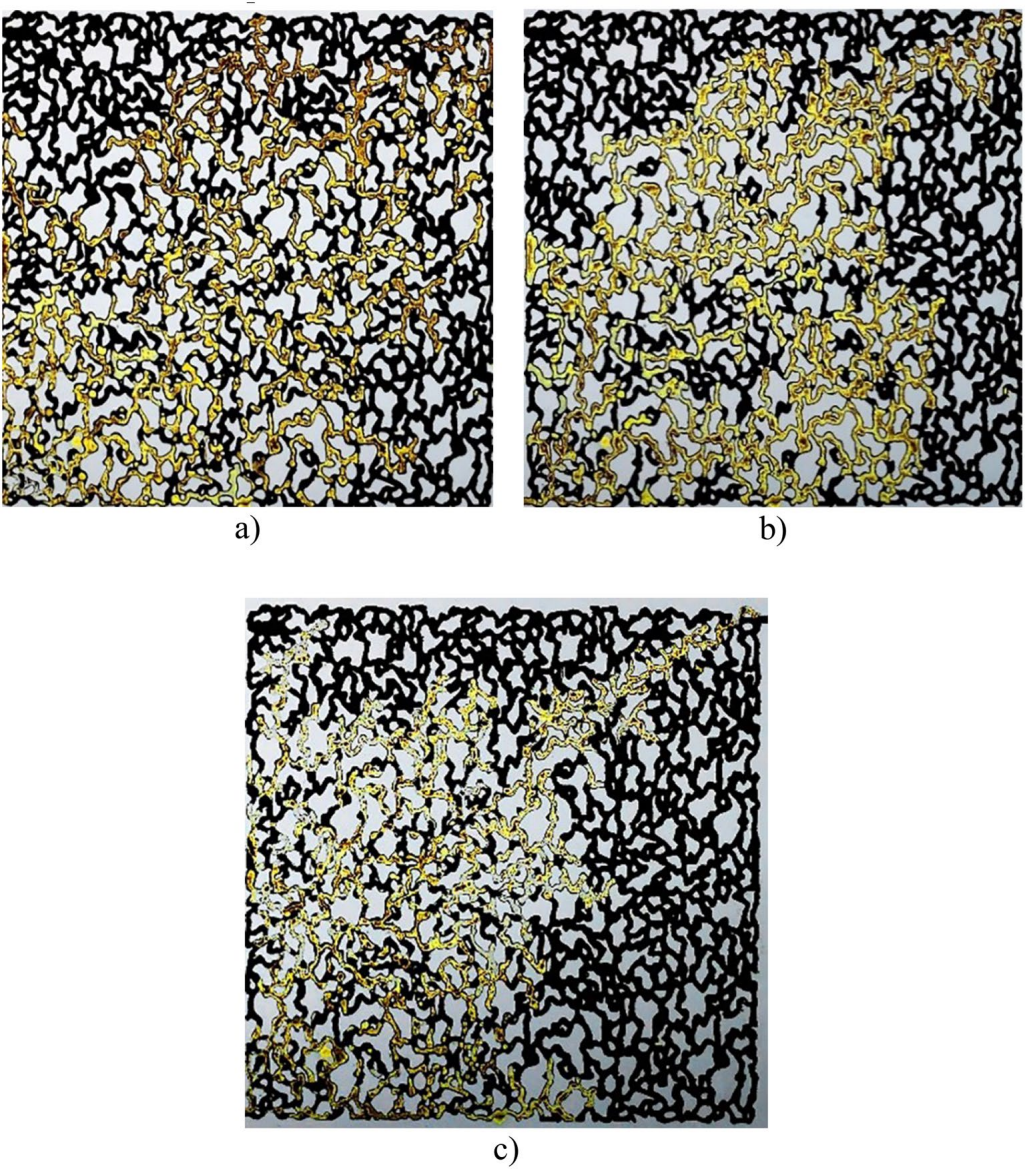
Microscopic images of oil displacement by different concentrations of CaCO_3_ (**a**) 0.01 wt.%, (**b**) 0.1 wt.%, (**c**) 0.5 wt.%.

In the case of CaCO_3_ nanoparticles, in Fig. 16, it is evident that the concentration of 0.01 wt.% recovered more oil because in this situation, IFT was at its minimum value but by increasing concentration, the area of oil which was swept by the nanofluid decrease. These results are in agreement with the IFT results in previous sections.

## Conclusions

This study consists of two parts. The first part studied the effect of SiO_2_ and CaCO_3_ nanoparticles in increasing the onset point of asphaltene precipitation. In the second part, their effect on oil recovery was investigated. Morphology studies of nano-inhibitors showed that CaCO_3_ nano-inhibitors have larger sizes and more regular structures after surface modification than SiO_2_ nano-inhibitors. SiO_2_ nano-inhibitors had better colloidal stability. However, both nano-inhibitors showed good stability based on qualitative and quantitative stability tests, especially in 4 h. The time of 4 h is vital because the micro model tests lasted for 4 h. Both nano-inhibitors recorded appropriate effects on increasing the onset point of asphaltene precipitation. However, CaCO_3_ nano-inhibitors had much better performance in lower concentrations. CaCO_3_ nano-inhibitors at a concentration of 0.01 wt.% increased the onset point of asphaltene precipitation up to 13% by volume percent of normal heptane. But in the concentration of 0.5 wt.%, the onset point has been recorded as 16.5%. In contrast, SiO_2_ nano-inhibitors increased the onset point of asphaltene precipitation from 10.75 to 16 when concentration increased from 0.01 to 0.5 wt.%. The optimum concentration of SiO_2_ and CaCO_3_ nanoparticles for reducing IFT occurred at 0.05 and 0.005 wt.%, respectively. They reduced the surface tension to 23.35 mN/m and 16.39 mN/m at these concentrations. Both nano-inhibitors have a favorable performance in changing wettability from oil-wet to water-wet. CaCO_3_ nano-inhibitor decreased the contact angle up to 27.31°, and SiO_2_ nanoparticles decreased up to 22.71° for the highest concentration of nanoparticles (0.5 wt.%). Based on wettability tests, CaCO_3_ nano-inhibitors in low concentrations (0.01 wt.%) have shown a lower contact angle and improved wettability. At this concentration, CaCO_3_ decreased the contact angle to 38.92°. But better performance was observed for the SiO_2_ nano-inhibitor at higher concentrations (22.71° at 0.5 wt.%). Both nano-inhibitors had a favorable effect in increasing oil recovery. The maximum oil recovery factor was 33% at 0.1 wt.% for SiO_2_ nanoparticles and 25% at 0.01 wt.% for CaCO_3_ nanoparticles. Thus, SiO_2_ nanoparticles performed better in increasing oil recovery than CaCO_3_ nanoparticles.

Finally, the limitations of this study include focusing on only two types of nanoparticles and conducting primarily laboratory-scale experiments. Future research should explore other nanoparticle types, conduct field-scale experiments, investigate temperature and pressure effects, utilize microscopic images, and optimize surface modification procedures for enhanced nanoparticle stability in reservoir conditions.

## Data Availability

All data generated or analyzed during this study are included in this article. Email for contact: ajafari@modares.ac.ir.
